# Risk factors for mortality of patients with ceftriaxone resistant *E. coli* bacteremia receiving carbapenem versus beta lactam/beta lactamase inhibitor therapy

**DOI:** 10.1186/s13104-019-4648-7

**Published:** 2019-09-23

**Authors:** Nosheen Nasir, Sara Ahmed, Samrah Razi, Safia Awan, Syed Faisal Mahmood

**Affiliations:** 1 Section of Adult Infectious Diseases, Dept. of Medicine, Aga Khan University, P.O. Box. 3500, Stadium Road, Karachi, 74800 Pakistan; 2 Aga Khan University Medical College, Karachi, Pakistan; 3 Aga Khan University, Karachi, Pakistan

**Keywords:** *Escherichia coli*, Carbapenem, Beta lactam/beta lactam inhibitor, Mortality

## Abstract

**Objective:**

Extended spectrum β-lactamases (ESBL) producing Enterobacteriaceae predominantly *E. coli* and *K. pneumoniae* bacteremia have limited treatment options and high mortality. The objective was to determine the risk factors for in-hospital mortality particularly treatment with carbapenem versus beta lactam/beta lactamase combination (BL/BLI) in patients with ceftriaxone resistant *E. coli* bacteremia. A retrospective cohort study was conducted at the Aga Khan University, Karachi, Pakistan. Adult patients with sepsis and monomicrobial ceftriaxone resistant *E. coli* bacteremia were enrolled. Factors associated with mortality in patients were determined using logistic regression analysis.

**Results:**

Mortality rate was 37% in those empirically treated with carbapenem compared to 20% treated with BL/BLI combination therapy (p-value: 0.012) and was 21% in those treated with a carbapenem compared to 13% in patients definitively treated with BL/BLI combination therapy (p-value: 0.152). In multivariable logistic regression analysis, only Pitt bacteremia score of ≥ four was significantly associated with mortality (OR: 7.7 CI 2.6–22.8) while a urinary source of bacteremia was protective (OR: 0.26 CI 0.11–0.58). In-hospital mortality in patients with Ceftriaxone resistant *E. coli* bacteremia did not differ in patients treated with either a carbapenem or BL/BLI combination. However, Pitt bacteremia score of ≥ 4 was strongly associated with mortality.

## Introduction

*Escherichia coli* is a leading cause of intra-abdominal, urinary tract, and bloodstream infections encountered in routine clinical practice [1]. It is a recognized pathogen for causing both community acquired and nosocomially acquired infections [1, 2]. Over the past two decades, antibiotic-resistant strains that produce extended spectrum β-lactamases (ESBL) have emerged among the Enterobacteriaceae, predominantly in *E. coli* and *Klebsiella pneumoniae* [2–4]. ESBLs are a group of hydrolyzing enzymes that provide resistance to third generation cephalosporins and aztreonam, but can in turn be hydrolyzed by clavulanic acid [5]. Several epidemiological studies have investigated the prevalence of ESBLs and their clinical impact [2, 3, 6]. There is considerable geographic variability in the prevalence of ESBLs with the prevalence being highest in from Latin America and Asia Pacific region followed by Europe and North America [4]. In a recently conducted meta-analysis from Pakistan, the pooled prevalence of ESBL producing Enterobacteriaceae was reported to be 40% [7]. This is similar to proportions reported from neighbouring countries of China [8] and India [9] and considerably higher than US and developed countries from Europe [5]. Hence, Pakistan is a country with high endemicity of community acquired ESBL producing Enterobacteriaceae with major public health implications [10, 11].

Since ESBL-producing organisms are frequently resistant to multiple antimicrobial agents therapeutic options for these infections are severely limited [12]. Moreover infections caused by ESBL producing organisms have been associated with high morbidity and mortality [13]. Studies have shown high Pitt bacteremia score and Charlson co-morbidity index to be predictive of death in patients with sepsis secondary to ESBL producing organisms [14]. Bacteremia with ESBL producing organisms has been associated with a delay in the initiation of appropriate treatment regimens and leads to an increase in the hospital length of stay and cost of overall treatment [14, 15]. Carbapenems have been considered as drugs of choice for treating severe infections caused by ESBL producing Enterobacteriaceae as they are not affected by ESBLs in vitro and clinical efficacy has been demonstrated in several observational studies as well as clinical trials [5, 16, 17]. A large multi-country open label non-inferiority randomized controlled (MERINO) trial which compared piperacillin tazobactam with meropenem as definitive treatment for Ceftriaxone resistant *E. coli* or *K. pneumoniae* bloodstream infection found significantly higher long term mortality in patients randomized to piperacillin tazobactam arm [16]. However, prolonged use of carbapenems places the community at risk of development of carbapenem resistance due to antibiotic selection pressure [18]. Thus it is imperative to find alternatives for treatment. Beta lactam/beta lactam inhibitor combinations (BL/BLI) have been widely studied both as empiric and definitive choice of antibiotics and while some studies have reported no difference in clinical outcomes with use of BL/BLI combination compared to carbapenem [19]; pooled data from meta-analysis have recommended their use for only clinically stable patients with urinary tract infections as the source [17].

Existing studies from Pakistan have shown that ESBL producing strains of Enterobacteriaceae show greater than 90 percent susceptibility for BL/BLI and hence may be an effective alternative [10, 11]. Carbapenem use has severe cost constraints in a low middle country like Pakistan and is reserved for very severe infections. Despite the high endemicity of ESBL producing organisms, data on risk factors for mortality and the optimal choice of antibiotic therapy is lacking. Our study aims to determine the risk factors for in-hospital mortality in patients with ESBL *E. coli* bacteremia using ceftriaxone resistance as a marker of ESBL presence; with emphasis on difference in outcome when patients were treated with a carbapenem compared to beta lactam/beta lactam inhibitor combination at a tertiary care hospital in Pakistan.

## Main text

### Methods

A retrospective cohort study was conducted at the Aga Khan University (AKU) Hospital in Karachi, Pakistan on patients admitted between January 2015 to December 2017. All adult patients with age greater than or equal to 18 years admitted with sepsis (based on established criteria) [20] and found to have monomicrobial ceftriaxone resistant *E. coli* bacteremia were included in the study. Patients were identified from the hospital information management system.

Data collected from medical records for all patients included age, sex, nosocomial or community-onset acquisition, type and severity of underlying conditions using the Charlson comorbidity index, source of bloodstream infection (BSI) according to clinical and microbiological data, Pitt bacteremia score, antimicrobial therapy, in-hospital mortality, and length of stay after BSI. The outcome measure was ‘In-hospital mortality’ and was defined as death due to all causes during same hospitalization. Antimicrobial therapy administered before susceptibility results were available was considered empirical and therapy administered afterwards was considered definitive.

Frequency and percentages were used to describe categorical variables and comparison between variables was determined using Chi square test or Fischer exact test where appropriate. p-value of < 0.05 was considered significant. Univariable analyses were performed to examine the effect of each variable on the in-hospital mortality. In univariable analysis; p-value < 0.15 was used as the level of significance in order not to exclude important variables from the model. Odds ratios (OR) and their 95% confidence intervals (CI) were estimated using logistic regression, with in-hospital mortality as an outcome. Data was analyzed using SPSS version 16.0.

This study was conducted after receiving exemption from ethical approval from Aga Khan University Ethical Review Committee (2734-MED-ERC-13).

### Results

Out of a total of the 295 patients that were included, 47% were males while 53% were females. The mean age of the patients was 57.71 years (SD: 17.16): Of the patients who died, the mean age was 55.3 years (SD: 17.9) years compared to 60.1 (SD: 16.5) years for those who survived (p-value: 0.046). The overall in-hospital mortality in patients with Ceftriaxone resistant *E. coli* bacteremia was 21%. The mean length of stay was 11.3 days (SD: 12.5) in those who died compared to 6.8 days (SD: 5.68) in those who survived (p-value < 0.001) (Table 1).

**Table 1:** Comparison of demographics and clinical features of patients with ceftriaxone resistant *E. coli* bacteremia who died compared to those who survived

Patient characteristics	Died (n = 62)	Recovered (n = 235)	p-value
Age (mean ± SD)	55.28 ± 17.87	60.14 ± 16.46	0.046
Gender			
Male	34	106	0.172
Female	28	129	
Charlson comorbidity index (CCI), mean (SD)	2.98 (2.12)	1.93 (1.73)	< 0.001
Pitt bacteremia score, median (IQR)	3 (4)	1 (2)	< 0.001
Site of infection			
Intra-abdominal	24	44	< 0.001
UTI	24	171	
CLABSI	4	4	
Gut translocation	4	5	
Source unclear	6	11	
Empirical antibiotics^a^			0.012
Carbapenem	20	33	
BL/BLI	31	121	
Definitive antibiotics^b^			
Carbapenem	36	138	0.152
BL/BLI	12	77	
Length of stay (mean ± SD)	11.27 ± 12.46	6.82 ± 5.68	< 0.001

^a^Empirical group includes only those patients who had been given either a carbapenem or BL/BLI combination treatment empirically

^b^Definitive group includes only those patients who had been given either a carbapenem or BL/BLI combination treatment as definitive treatment

In the univariate analysis to determine association between choice of empirical treatment and mortality; out of 60 patients who died, n = 20 had received a carbapenem and n = 31 had received a BL/BLI combination therapy. Mortality rate was 37% in those treated with carbapenem compared to 20% in patients empirically treated with BL/BLI combination therapy (p-value: 0.012). For definitive treatment; n = 36 received a carbapenem whereas n = 21 received BL/BLI combination therapy among those who died. Mortality rate was 21% in those treated with a carbapenem compared to 13% in patients definitively treated with BL/BLI combination therapy (p-value: 0.152). Other variables found to be significantly associated with mortality included increasing mean Charlson comorbidity index and increasing Pitt bacteremia score (Table 1).

However, in multivariable logistic regression analysis; a Pitt bacteremia score of greater than or equal to 4 was significantly associated with mortality (OR: 16.8 CI 7.47–38.0) while presence of urinary tract infection being the source of bacteremia was protective (OR: 0.33 (CI 0.15–0.73)) after adjusting for confounding from Charlson comorbidity index of greater than or equal to 3 (Table 2). Neither the empirical nor definitive antibiotic therapy with carbapenem versus BL/BLI combination therapy was significantly associated with in-hospital mortality.

**Table 2:** Multivariable analysis showing factors associated with in-hospital mortality in patients with Ceftriaxone resistant *E. coli* bacteremia

Variables	aOdd ratio (95% CI)	*p*-value
Pitt bacteremia score		
< 4 (Ref)	1.0	< 0.001
≥ 4	16.85 (7.47–38.0)	
Source of infection		
Intra-abdominal (Ref)	1.0	0.002
Urinary tract infection	0.33 (0.15–0.73)	0.006
CLABSI	3.03 (0.62–14.66)	0.16
Gut translocation	2.97 (0.50–17.43)	0.22
Source unclear	1.07 (0.28–4.03)	0.91
Charlson comorbidity index score		
< 3 (Ref)	1.0	0.055
≥ 3	1.98 (0.98–3.97)	

### Discussion

Empiric and definitive choice of antimicrobial therapy for ESBL producing Enterobacteriaceae has been subject of debate since a long time because of the implications of using carbapenem for ESBL infections which while effective has the potential for promoting carbapenem resistance [18]. Our study did not show an association of mortality with use of either a carbapenem or a BL/BLI combination treatment in ceftriaxone resistant *E. coli* bacteremia whether it was used empirically or definitively. These findings are similar to a post hoc analysis of prospective cohort conducted by Rodriguez-Bano et al. [19] where neither carbapenem nor BL/BLI combination influenced mortality or hospital length of stay. However, the recently conducted multi-country open label non-inferiority randomized controlled (MERINO) trial which compared piperacillin tazobactam with meropenem as definitive treatment for Ceftriaxone resistant *E. coli* or *K. pneumoniae* bloodstream infection found significantly higher long term mortality in patients randomized to piperacillin tazobactam arm and concluded that piperacillin tazobactam was not non-inferior (risk difference: 8.6%; p = 0.90). The authors therefore recommended avoiding use of this combination for Ceftriaxone resistant bloodstream infections [16]. However, the trial had several limitations owing to its pragmatic design with crossover in both arms and that the cause of 30-day mortality was unrelated to infection. Despite the criticism, the results were quite robust for the conclusion [21]. Given the conclusion of this trial, the concern for increased carbapenem resistance with increasing use has left many experts to consider piperacillin tazobactam for less severe infections with ESBL producing enterobacteriaceae [21]. Despite this, similar to the MERINO trial, our study showed that the short-term outcomes (i.e. inpatient mortality) is similar for both carbapenems and BL/BLI combinations in ceftriaxone resistant Enterobacteriaceae. Our study was however not designed to look at the long term effects between the two regimens, including the outcomes post discharge. As for other comparisons between both antibiotics as empirical therapy, there has been a systematic review and meta-analysis of non-randomized studies which concluded that carbapenems should be given preference as drug of choice in patients with ESBL-producing Enterobacteriaceae bloodstream infections [17]. However, there was substantial clinical and statistical heterogeneity in the studies included in this review and a more recent review conducted by Sfeir et al. [22] in 2018 have not found any difference in 30-day mortality with use of either carbapenem or BL/BLI combination. Comparison studies for appropriate choice of antibiotic for ESBL producing Enterobacteriaceae bacteremia are lacking from South Asian region which differs from the west in terms of geographical and economic diversity. A resource poor country like ours where carbapenem use has severe cost implications and is considered to be one of the last resort antibiotics, it is imperative to determine which antibiotic is better and we recommend future randomized controlled trials in this region based on our results.

Several predictors of mortality; apart from choice of antibiotics; have been identified in various studies for ESBL Enterobacteriaceae bloodstream infections. These include high Charlson comorbidity index, high Pitt bacteremia scores and source of bloodstream infection other than urinary tract [13, 14, 23]. In our study also we found significant association of Pitt bacteremia score of greater than 4 to be significantly associated with in-hospital mortality in patients with ceftriaxone resistant *E. coli* bacteremia. Moreover, similar to findings of other studies, presence of urinary tract infection was favourable.

### Conclusion

In conclusion, our study shows that there was no difference in in-hospital mortality in patients with ceftriaxone resistant *E. coli* bacteremia in patients treated with either a carbapenem or BL/BLI combination whether empirically or definitively. However, Pitt bacteremia score of greater than or equal to 4 was strongly associated with mortality while urinary tract as site of infection was protective. We recommend randomized controlled trials for further delineating choice of appropriate antibiotic in our resource poor region.

## Limitations

There are several limitations of our study. Firstly, we have used Ceftriaxone resistance as a marker of ESBL presence and we do not have genotypic and MIC data for our isolates. However, the study is intended to guide in real life scenarios encountered in clinical practice in our setting where molecular studies are not widely available. Moreover, large pragmatic trial has also utilized ceftriaxone resistance as surrogate marker for better feasibility [16]. Secondly, our study is a single center study and hence the generalizability may be limited to our region. Similarly, the lack of difference in mortality, especially between the two treatment regimens, may have been due to the small sample size.

## Data Availability

All data generated or analysed during this study are included in this published article.

## Abbreviations

ESBL: extended spectrum β-lactamases
BL/BLI: beta lactam/beta lactamase combination
BSI: bloodstream infection
ERC: Ethical Review Committee
